# 국내 치매안심센터를 이용하는 치매노인 가족돌봄자의 돌봄부담 영향요인: 횡단적 서술적 조사연구

**DOI:** 10.7586/jkbns.24.025

**Published:** 2024-11-21

**Authors:** Ja Eun Kim, Soo Jin Lee

**Affiliations:** 1 Department of Nursing, Gangdong University, Eumsung-gun, Chungbuk, Korea; 1 강동대학교 간호학과; 2 Department of Nursing, Korea National Open University, Seoul, Korea; 2 한국방송통신대학교 간호학과

**Keywords:** Aged, Caregiver burden, Dementia, Family, 노인, 돌봄부담, 치매, 가족

## Abstract

**Purpose:**

This study aimed to identify factors influencing the care burden faced by family caregivers of older adults with dementia who use dementia care centers.

**Methods:**

This study investigated the care burden, family caregiver factors, care-recipient factors including behavioral and psychological symptoms of dementia (BPSD), depression levels, dementia attitudes, and resilience. Data were collected from March 15 to September 30, 2022. In total, 234 family caregivers using dementia care centers in five districts in Seoul, Korea, participated. Data were analyzed using the t-test, analysis of variance, Pearson correlation coefficients, and multiple regression analysis with SPSS/Windows version 27.0.

**Results:**

The mean score for care burden was 22.97. The factors influencing the care burden were BPSD (ß = .41, *p* < .001), depression levels (ß = .26, *p* < .001), hours of caregiving (ß = .18, *p* < .001), and resilience (ß = -.12, *p* = .022). The explanatory power of the final model was 46.1% (F = 47.76, *p* < .001).

**Conclusion:**

This study provides valuable insights into the care burden faced by family caregivers. Based on these results, programs for family caregivers should be developed with consideration of BPSD in older adults with dementia. Additionally, interventions that relieve depression and strengthen the resilience of family caregivers should be prioritized. Furthermore, it is essential to extend support systems to accommodate caregivers’ time constraints.

## 서론

### 1. 연구의 필요성

대한민국 치매 현황 2022 보고서에 따르면 65세 이상 치매 환자 수는 약 89만 명으로 65세 노인 인구 858만 명의 10.4%를 차지하고 있으며, 2040년에는 226만 명이 넘을 것으로 예상하고 있다[1]. 이러한 치매노인 수와 연령의 증가는 사회·경제적으로 많은 영향을 미친다. 특히, 우리나라 치매 관리 비용은 2021년 한 해 동안 18조 7천억 원(국내 총생산의 약 0.9%)이었으며, 2040년에는 약 71조 8천억 원까지 증가할 것으로 추정하고 있다[1].

이러한 치매 유병률 증가는 지역사회 역할 강화로 이어지는데, 치매 환자 대부분이 친숙하고, 독립적·통제적이며, 자신의 정체성을 촉진할 수 있는 환경인 집에 머물며 치료를 이어가길 원하기 때문이다. 그리고 경제협력개발기구(Organization for Economic Cooperation and Development)에 속한 국가들도 가능하면 치매 환자가 지역사회에 오래 머물도록 권장하고 있다[2]. 이는 지역사회 내에서 치료가 제공될 때 더 양질의 삶을 제공하고 비용도 절약할 수 있기 때문이며, 현재는 치매 환자가 건강을 유지하며 장기간 지역사회 내에 거주할 수 있도록 치매 친화적인 커뮤니티를 만드는 것에 중점을 두고 있다[2]. 우리나라 역시 지역사회 거주 치매노인의 비율이 2018년 76.7%에서 2020년 85.1%로 지속해서 증가하고 있으며[3], 정부에서도 치매 환자가 본래 살던 지역사회에 거주할 수 있도록 하는 데 목적을 둔 제4차 치매관리종합계획을 추진하고 있고, 비공식적인 돌봄 자원인 가족이 많은 부분을 부담하고 있다[4].

가족돌봄자는 치매노인 돌봄으로 인해 일상생활 뿐만 아니라, 신체적, 정서적, 경제적으로 많은 영향을 받는다[4]. 특히, 치매는 단기 기억장애로 시작하여 지남력 상실, 정신장애, 언어장애, 행동장애 등을 초래하는 질환으로[5] 질병이 진행됨에 따라 인지기능 저하, 일상생활 수행 능력 저하, 행동심리증상 등이 나타나게 되어 타인에게 의존하는 정도가 높아진다[6]. 따라서, 가족돌봄자는 치매노인의 중증도가 높아질수록 돌봄을 위한 고도의 기술과 시간이 필요하게 된다. 즉, 가족돌봄자는 장기간의 돌봄부담으로 긴장, 분노, 우울, 불쾌감 뿐만 아니라, 신경정신과적 질환도 경험할 수 있다[7]. 게다가, 장기간의 돌봄부담은 만성 스트레스로 작용하여 시상하부-뇌하수체-부신 축에 의해 스트레스 호르몬 분비를 증가시키게 되고 반복적인 각성과 신체 부담은 병리적 변화를 유발할 수 있다[8]. 또한, 돌봄부담으로 인한 심리적 고통은 불량한 식습관, 운동 부족 등을 유발하여 심혈관, 대사, 면역 기능 등에 악영향을 미치기도 한다[8].

돌봄부담에 대한 선행연구에 따르면, 치매노인 돌봄자 대부분(75%)은 높은 수준의 돌봄부담을 경험하고 있다[1]. 그리고 돌봄부담 영향요인은 치매노인 관련 특성과 가족돌봄자 관련 특성으로 구분된다. 먼저 치매노인이 남성이며, 나이가 많고, 교육 수준이 높을 때, 행동심리증상이 많을 때, 치매 중증도가 높을 때, 일상생활 의존도가 높을 때 돌봄부담이 증가하였다[6]. 그리고 가족돌봄자의 나이가 많고, 건강상태가 좋지 않을 때, 돌봄 기간이 5년 이상일 때, 돌봄시간이 길 때, 교대할 사람이 없을 때, 주관적 건강상태가 나쁠 때 돌봄부담이 높아지는 것으로 나타났다[9-12].

게다가, 돌봄부담은 가족돌봄자의 우울, 치매태도, 회복탄력성의 영향도 받는다. 가족돌봄자의 우울 증상 발생 비율은 23%~85%이고, 이는 일반인보다 2~3배 높은 수치이다[13]. 또한, 지역사회 건강조사[14]에 따르면, 치매환자를 가정에서 돌보는 가족의 우울 증상 양성률은 4.4%로 증상이 없는 경우보다 1.9% 더 높았으며, 우울과 돌봄부담은 양의 상관관계에 있다[7]. 반면, 치매태도와 회복탄력성은 돌봄부담을 낮추는 역할을 하는 것으로 알려져 있다. 치매태도는 치매노인에 대한 돌봄자의 태도를 의미하는 것으로, 치매태도에 따라 대처와 치료 활동이 달라지며, 선행연구에서는 치매태도가 긍정적일수록 돌봄부담이 감소함을 보였다[15,16]. 그리고 회복탄력성은 스트레스나 역경에도 원상태로 회복하고 성공적으로 적응할 수 있는 능력을 의미하며, 선행연구 결과 회복탄력성이 높을수록 돌봄부담이 낮아지는 것으로 나타났다[17]. 하지만, 회복탄력성을 포함한 치매노인 가족돌봄자에 대한 연구는 극히 적으며, 치매노인 가족돌봄자의 우울, 치매태도, 회복탄력성이 돌봄부담과 어떠한 관계가 있는지를 밝히는 연구도 많지 않다.

돌봄부담이 높아지면 가족의 전반적인 기능이 약화되고, 가족돌봄자의 삶의 질이 떨어지며, 스트레스와 우울이 증가하여 돌봄의 질을 떨어뜨리게 된다[1,18]. 그리고 치매 증상이 심해지면 추가적인 서비스를 이용하게 되는데, 이는 경제적 부담으로 작용한다[4]. 반면, 치매노인 가족의 치매 극복 수기 분석 결과에 따르면, 가정에서 치매노인을 돌보는 가족들은 돌봄으로 인해 좌절과 고통을 경험하지만, 그와 동시에 돌봄을 긍정적으로 승화시키기도 한다[19]. 따라서, 돌봄부담 영향요인 분석 시, 부정적 요인과 긍정적 요인 모두를 포함한 접근이 필요하다[20].

이에 본 연구는 치매노인 가족돌봄자의 돌봄부담, 우울, 치매태도, 회복탄력성 수준을 파악하고, 연구변수 간의 관계와 돌봄부담 영향요인을 확인하고자 한다. 그리고 향후 치매노인 가족돌봄자의 돌봄부담을 줄이고 더 잘 적응할 수 있도록 도움이 되는 제도 및 상담 방안을 수립하며, 개선하는 데 필요한 기초자료로 활용하고자 한다.

### 2. 연구 목적

본 연구의 목적은 치매노인 가족돌봄자의 돌봄부담에 영향을 미치는 요인을 규명하는 데 있다. 구체적인 연구 목적은 다음과 같다.

∙가족돌봄자와 치매노인의 일반적 특성을 파악한다.

∙가족돌봄자의 돌봄부담, 우울, 치매태도, 회복탄력성을 확인한다.

∙가족돌봄자의 일반적 특성에 따른 돌봄부담의 차이를 파악한다.

∙가족돌봄자의 돌봄부담, 우울, 치매태도, 회복탄력성의 상관관계를 파악한다.

∙가족돌봄자의 돌봄부담에 영향을 미치는 요인을 확인한다.

## 연구 방법

### 1. 연구 설계

본 연구는 지역사회 치매안심센터에 등록하여 센터의 서비스를 이용하며, 지역사회에서 치매노인을 돌보고 있는 가족돌봄자의 돌봄부담에 영향을 미치는 요인을 분석하고 확인하기 위한 서술적 조사연구이다. 이 연구는 Strengthening the Reporting of Observational Studies in Epidemiology 보고 지침(https://www.strobe-statement.org)에 따라 기술하였다.

### 2. 연구 대상

본 연구의 대상자는 서울시 치매안심센터를 이용 중인 치매노인의 가족돌봄자이다. 연구대상자는 치매 노인을 3개월 이상 돌보는 가족 중, 만 19세 이상인 주 부양자 1인을 선정하여 조사하였고, 요양원이나 요양병원 등 시설에 입소한 치매 노인의 가족은 제외하였다.

연구대상자 수는 G*Power 3.1.9.7 프로그램을 이용하여 산출하였다[21]. 표본수 산출 시 선행연구를 참고하였으며[11,22], 돌봄부담의 영향요인을 파악하기 위한 다중회귀분석에 필요한 대상자 수는 효과 크기 0.15, 검정력 1-β = .95, 유의수준 α = .05, 예측변수 18개로 하였을 때 213명이 산출되었다. 탈락률을 고려하여 총 247부를 모집하였으며, 불완전한 응답을 한 13부를 제외한 234부가 최종 분석에 사용되었다.

### 3. 연구 도구

#### 1) 가족돌봄자의 일반적 특성

본 연구에서는 가족돌봄자의 일반적 특성으로 가족돌봄자의 성별, 연령, 교육 수준, 종교 여부, 주관적 건강상태 등의 인구사회학적 특성과 치매노인과의 관계, 동거 여부, 돌봄시간, 돌봄 기간 등의 돌봄 관련 특성을 조사하였다.

#### 2) 치매노인의 일반적 특성

치매노인의 일반적 특성으로는 치매노인의 성별, 연령, 교육 수준 등의 인구사회학적 특성과 행동심리증상을 조사하였다. 치매노인의 행동심리증상은 대한노인정신의학회에서 한국어판으로 번안하고 Kim 등[23]이 표준화한 신경정신행동 검사-간편형(Neuropsychiatric Inventory-Questionnaire, NPI-Q)를 사용하였다. 도구는 치매노인의 12가지 행동심리증상의 심한 정도와 가족고통 정도를 평가하도록 구성되어 있다. 본 연구에서는 치매노인의 행동심리증상 파악을 목적으로 본 도구를 사용하였으므로, 가족고통 정도를 제외한 행동심리증상의 심한 정도만 분석에 활용하였다. 행동심리증상의 심한 정도는 치매노인의 행동심리증상을 ‘없음(0점)’, ‘경함(1점)’, ‘보통(2점)’, ‘심함(3점)’으로 점수화한 후 이를 합산하여 평가하며, 총점이 높을수록 증상이 심함을 의미한다. 한국어판 NPI-Q는 Cronbach’s α = .85 이었으며[23], 본 연구에서는 Cronbach’s α = .95이었다.

#### 3) 가족돌봄자의 돌봄부담

돌봄부담은 Zarit 등[24]의 ‘자릿 부양 부담 평가(Zarit Burden Inventory)’를 Bédard 등[25]이 단축형 척도로 개발한 도구를 Kim 등[26]이 한국어로 번안 및 검증한 ‘단축형 자릿 부양 부담 평가(Short Zarit Burden Inventory)’를 사용하였다. 도구는 총 12개 문항으로 개인 부담(9문항)과 역할 부담(3문항)으로 구성된다. 각 문항은 ‘전혀 없다(0점).’, ‘거의 없다(1점).’, ‘가끔 있다(2점).’, ‘자주 있다(3점).’, ‘항상 있다(4점)’로 점수화하며, 총점이 높을수록 돌봄부담이 큰 것을 의미한다. Kim 등[26]의 연구에서 개인 부담은 Cronbach’s α = .88, 역할 부담은 Cronbach’s α = .78이었다. 본 연구에서 Cronbach’s α = .91이었고, 하위 범주인 개인 부담은 Cronbch’s α = .93, 역할 부담은 Cronbch’s α = .71이었다.

#### 4) 가족돌봄자의 우울

가족돌봄자의 우울 정도는 Spitzer 등[27]이 개발한 도구를 Park 등[28]이 한국어판으로 번역하여 신뢰도와 타당도를 검증한 한글판 우울증 선별 도구(Patient Health Questinnaire-9)를 사용하였다. 도구는 총 9문항으로 각 항목에 따른 증상 정도를 0∼3점으로 점수화한 후, 합산하여 우울 정도를 판단한다. 우울 여부를 판단하는 절단점(cut-off point)은 10점이며, 세부적으로 0~4점 우울 아님, 5~9점 가벼운 우울, 10~19점 중간 정도 우울, 20~27점 심한 우울로 판단할 수 있다. 한국어판 개발 당시 신뢰도는 Cronbach’s α = .81이었고[28], 본 연구에서는 Cronbach’s α = .89이었다.

#### 5) 가족돌봄자의 치매태도

치매태도는 O’Connor와 McFadden [29]가 개발한 치매태도 척도(Dementia Attitude Scale)를 Choi 등[15]이 지역사회 치매노인 돌봄자를 대상으로 개발한 한국어판 도구를 사용하였다. 도구는 총 20문항이며, 인지적인 측면을 평가하는 지식소척도(10문항)와 정서 및 행동을 평가하는 안정소척도(10문항)로 구성된다. 각 문항은 ‘전혀 그렇지 않음’을 1점, ‘매우 그러함’이 7점으로 점수화하고, 총점이 높을수록 치매에 긍정적 태도를 보이고 있음을 의미한다. 한국어판 개발 당시 도구의 신뢰도는 Cronbach’s α = .83∼.85이었다[15]. 본 연구에서 Cronbach’s α = .77이었고, 하위범주인 지식소척도는 Cronbach’s α = .77, 안정소척도는 Cronbch’s α = .73이었다.

#### 6) 가족돌봄자의 회복탄력성

회복탄력성은 Smith 등[30]이 개발한 단축형 회복탄력성(Brief Resilience Scale)을 Choi 등[31]이 한국어로 번안한 도구를 저자의 승인을 받은 후 사용하였다. 도구는 스트레스에서 회복할 수 있는 능력을 평가하며, 총 6개 문항으로 구성되었다. 각 문항은 ‘전혀 그렇지 않다’ 0점부터 ‘매우 그렇다’ 5점까지로 점수화하며, 총점이 높을수록 회복탄력성이 높은 것을 의미한다. 한국어판의 신뢰도는 Cronbach’s α = .75이었고[31], 본 연구에서는 Cronbach’s α = .86이었다.

### 4. 자료 수집

본 연구는 서울시 총 25개 구 치매안심센터 중, 5개 구 치매안심센터에 등록된 치매노인의 가족돌봄자를 대상으로 하였고, 자료는 2022년 3월 15일부터 9월 30일까지 약 6개월 동안 수집하였다. 연구자는 서울시 5개 구 치매안심센터의 총괄팀장에게 본 연구의 목적, 내용, 방법에 대하여 설명한 후 협조받았고, 설문을 시행하기 전, 치매안심센터 팀장이 설문조사를 담당할 각 센터의 담당자들에게 본 연구의 목적, 내용, 방법을 사전교육하였다. 설문지 작성은 20분 정도 소요되었으며, 대부분 연구대상자 본인이 직접 작성하였다. 그리고 설문지를 직접 작성하기 어려운 경우(n = 15)에는 사전교육을 받은 설문조사 담당자가 설문 내용을 읽어주어 작성할 수 있도록 하였다.

### 5. 자료 분석

수집된 자료는 SPSS 27.0 (IBM Corp., Armonk, NY, USA) 프로그램을 사용하여 분석하였다. 통계적 유의성은 *p* = .05로 하였다. 구체적인 자료 분석 방법은 다음과 같다.

∙가족돌봄자와 치매노인의 일반적 특성, 돌봄부담, 우울, 치매태도, 회복탄력성은 기술통계(빈도, 백분율, 평균, 표준편차)로 분석하였다.

∙가족돌봄자의 일반적 특성에 따른 돌봄부담의 차이는 t-test와 ANOVA로 분석하였고, 사후분석은 Scheffé́ test로 하였다.

∙가족돌봄자의 일반적 특성, 돌봄부담, 우울, 치매태도, 회복탄력성의 상관관계는 Pearson correlation coefficients를 이용하였다.

∙가족돌봄자의 돌봄부담 영향요인을 확인하기 위해 단계적 다중회귀분석(stepwise multiple regression analysis)을 시행하였다.

### 6. 윤리적 고려

본 연구는 한국방송통신대학교 생명윤리심의위원회(IRB)의 심의를 받은 후 자료 수집을 진행하였다(IRB No. ABN01-201102-21-03). 설문조사는 본 연구 목적을 이해하고 자발적으로 동의한 가족돌봄자를 대상으로 하고 서면 동의서를 받은 후 진행하였다. 수집된 자료는 학문적 연구 목적 외에 다른 목적으로 사용되지 않으며 본인이 원하면 언제든지 연구를 철회할 수 있음을 설명하였고, 궁금한 사항은 언제든지 연구자에게 문의하도록 하였다. 그리고 수집된 개인정보는 개인정보 보호법에 따라 암호화한 후 관리하고, 연구 종료 후 5년간 보관되며 이후 완전히 폐기될 것임을 설명하였다. 연구대상자와 설문조사 담당자들에게는 설문이 완료된 후, 소정의 답례품을 제공하였다.

## 연구 결과

### 1. 가족돌봄자 요인

가족돌봄자는 대부분 여성(194명, 82.9%)이었고, 평균 연령은 65.8세(± 12.83)였으며, 65∼75세(76명, 32.5%)가 가장 많았다. 주관적 건강상태는 보통(93명, 39.7%), 나쁜 편(72명, 30.8%), 건강한 편(69명, 29.5%)인 순으로 나타났다(Table 1). 치매노인과의 관계는 배우자(123명, 52.6%)와 자녀(82명, 35.0%)가 대부분이었고, 치매노인과 동거 상태(192명, 82.1%)인 경우가 월등히 높았다. 돌봄시간은 하루 평균 13.62시간(± 7.80)으로, 12시간 이상(127명, 54.3%)이 더 많았다. 돌봄 기간은 평균 4.26년(± 3.36)이었고, 5년 이하가 71.8%(168명)이었다.

### 2. 치매노인 요인

치매노인은 여성(115명, 49.1%), 남성(119명, 50.9%)으로 비슷한 분포를 나타냈으며, 연령은 평균 81.7세(± 7.71)로 55세부터 103세까지 분포하였다. 치매노인 중 91.5%에게서 행동심리증상이 나타났으며, 행동심리증상의 심한 정도는 평균 9.66점(± 7.50)이었다(Table 1).

### 3. 가족돌봄자의 돌봄부담, 우울, 치매태도, 회복탄력성

가족돌봄자의 돌봄부담은 평균 22.97점(± 9.95)이었다. 그리고 하위범주인 개인 부담은 평균 17.12점(± 8.56)이었으며, 역할 부담은 평균 5.81점(± 2.64)이었다. 우울 정도는 평균 7.07점(± 5.99)이었다. 가벼운 우울(27.4%)이 가장 많았고, 다음은 중등도 우울(26.9%)로 나타났다. 10점을 절단점(cut-off point)으로 하였을 때 10점 이상인 중등도 우울과 심한 우울은 30.8%(72명)이었다. 치매태도는 평균 83.03점(± 16.20)이었고, 치매태도의 하위범주인 지식소척도는 평균 42.40점(± 10.20), 안정소척도는 평균 40.63점(± 10.39)이었다. 그리고 회복탄력성은 평균 19.55점(± 4.72)이었다(Table 2).

### 4. 가족돌봄자 요인과 치매노인 요인에 따른 돌봄부담 차이

가족돌봄자 요인에 따른 돌봄부담 차이를 분석한 결과, 돌봄부담은 주관적 건강상태(F = 10.65, *p* < .001), 동거 여부(t = 2.64, *p* = .009), 돌봄시간(t = 3.96, *p* < .001)에서 통계적으로 유의한 차이가 있었으며, 주관적 건강상태가 나쁜 경우, 치매노인과 동거상태인 경우, 돌봄시간이 12시간 이상인 경우에 돌봄부담 점수가 더 높았다(Table 1). 그리고 치매노인 요인에서는 행동심리증상에서 통계적으로 유의한 차이가 있었고(t = −5.63, *p* < .001), 행동심리증상이 있는 경우에 돌봄부담 점수가 더 높았다(Table 1).

### 5. 주요 변수의 상관관계

주요 변수 간의 관계를 분석하고자 피어슨 상관관계 분석을 하였다. 상관관계 분석에는 돌봄시간, 행동심리증상의 심한 정도, 돌봄부담, 우울 정도, 치매태도, 회복탄력성이 포함되었으며, 그 결과는 Table 3과 같다. 돌봄부담은 가족돌봄자의 돌봄시간(r = .28, *p* < .001), 행동심리증상의 심한 정도(r = .56, *p* < .001), 우울 정도(r = .52, *p* < .001)와 통계적으로 유의한 정적인 상관관계를, 치매태도(r = −.25, *p* < .001), 회복탄력성(r = −.34, *p* < .001)과는 통계적으로 유의한 부적인 상관관계를 보였다. 그리고 돌봄부담의 하위범주인 개인 부담에서도 유사한 결과가 나타났다. 하지만, 역할 부담은 돌봄시간을 제외한 나머지 변수에서만 통계적으로 유의한 상관관계를 보였다.

### 6. 가족돌봄자의 돌봄부담 영향요인

가족돌봄자의 돌봄부담에 영향을 미치는 요인을 확인하기 위해 변수를 단계적으로 투입하여 다중회귀분석(stepwise multiple regression)을 실시하였다. 투입된 변수는 돌봄부담과 통계적으로 유의한 차이 또는 유의한 상관관계를 나타낸 주관적 건강상태, 동거 여부, 돌봄시간, 행동심리증상의 심한 정도, 우울 정도, 치매태도, 회복탄력성이었다. 분석 시, 범주화 변수인 주관적 건강상태는 더미 변수로 변경한 후 분석하였다.

단계적 다중회귀분석 결과, 회귀모형은 통계적으로 유의하게 나타났으며(F = 47.76, *p* < .001), 회귀모형의 설명력은 약 46.1%로 나타났다(Table 4). Durbin-Watson 통계량은 1.96으로 2에 근사한 값을 보여 잔차의 독립성 가정에 문제가 없는 것으로 평가되었고, 분산팽창지수(variance inflation factor)도 모두 10 미만으로 작게 나타나 다중공선성 문제도 없는 것으로 판단되었다. 다중회귀분석의 유의성 검증 결과, 행동심리증상의 심한 정도(β = .41, *p* < .001), 우울(β = .26, *p* < .001), 돌봄시간(β = .18, *p* < .001), 회복탄력성(β = -.12, *p* = .022)의 순으로 돌봄부담에 영향을 미치는 것으로 나타났다. 그리고 가족돌봄자의 돌봄부담은 치매노인의 행동심리증상이 심할수록, 가족돌봄자의 우울 정도가 높을수록, 돌봄시간이 많을수록, 회복탄력성 점수가 낮을수록 증가하였다.

## 논의

본 연구는 지역사회에서 치매노인을 돌보는 가족돌봄자의 돌봄부담에 영향을 미치는 요인을 규명하고자 수행되었다. 이를 위해 치매노인의 일반적 특성과 가족돌봄자의 일반적 특성, 돌봄부담, 우울, 치매태도, 회복탄력성을 조사하였으며, 연구결과를 논의하면 다음과 같다.

본 연구의 치매노인은 남녀 유사한 비율이었고, 나이는 평균 81.7세였다. 행동심리증상은 한 개 이상 있는 경우(91.5%)가 대부분이었으며, 행동심리증상의 심한 정도는 평균 9.66점이었다. 이는 중앙치매센터[3]의 치매노인 연령별 분포에서 80세 이상이 63.4%인 결과와 유사하였고, 행동심리증상의 심한 정도는 10.72점으로 나타난 선행연구[22] 결과보다 다소 낮은 점수를 보였다.

본 연구의 가족돌봄자는 나이가 평균 65.8세였고, 75세 이상이 26.9%이었다. 치매노인과의 관계는 배우자 또는 자녀인 경우가 많았고, 하루 돌봄시간은 평균 13.62시간으로 돌봄에 12시간 이상을 할애하는 경우가 54.3%였다. 주관적 건강상태는 대부분 보통(39.7%)이었지만, 나쁜 경우도 약 30%를 차지하였다. 이는 다수의 선행연구 결과와 유사한데[13,22,32], 가족돌봄자의 평균 나이는 61~66세였으며, 배우자 또는 자녀가 대부분이었고, 하루 돌봄시간은 9~14시간이었다. 그리고 주관적 건강상태 역시 보통과 나쁜 경우의 비율이 38.2%, 36.7%로 유사하였다[32]. 즉, 치매노인, 가족돌봄자 모두 고령화되는 추세이고, 치매노인 돌봄에 시간 대부분을 할애하고 있어 건강상태도 직·간접적으로 영향을 받고 있었다.

연구결과 가족돌봄자의 돌봄부담은 22.97점으로, 치매노인에게 행동심리증상이 있을 때, 가족돌봄자의 주관적 건강상태가 나쁠 때, 치매노인과 동거할 때, 하루 돌봄시간이 12시간 이상일 때 돌봄부담이 유의하게 더 높았는데, 이는 이미 많은 선행연구에서도 유사하게 보고되고 있다[9,10,12,17,33]. 특히, 본 연구를 비롯해 선행연구[4]의 가족돌봄자 70%~80%는 치매노인과 동거를 하고 있어 자연스럽게 하루 6~12시간 돌봄을 제공하게 되었고, 이들에게 개인 시간은 거의 없었다. 돌봄시간은 돌봄을 측정하는 객관적 지표로 사용될 만큼 돌봄에 있어서 중요한 부분을 차지한다[34]. 하지만, 과도한 돌봄시간과 기간의 장기화는 가족돌봄자의 소진, 건강 악화 등의 부정적 영향을 미칠 뿐만 아니라, 돌봄부담을 단시간 내에 심화시킬 수 있으므로 주의가 필요하다[11]. 또한, 과도한 돌봄부담은 치매노인에 대한 학대를 초래하거나, 요양 시설로 조기에 입소시키는 원인이 될 수 있다는 점도 염두에 둘 필요가 있다. 따라서, 치매노인을 돌보는 가족의 보호 기능이 최대한 건강하게 오래 유지될 수 있도록 지역사회 내의 다양한 지지 체계가 필요함을 알 수 있는 결과이다.

본 연구의 가족돌봄자는 가벼운 우울(27.4%)이 가장 많았고, 중등도 이상 우울은 30.8%이었으며, 돌봄부담과 유의한 양의 상관관계를 보였다. 또한, 선행연구에서도 가족돌봄자의 우울 비율은 23%~35%로 일반인보다 2~3배 높았고[13], 우울 정도가 높을수록 돌봄부담이 증가하는 것으로 나타났다[7]. 그리고 치매노인을 돌보는 것은 치매가 없는 노인을 돌보는 것보다 더 높은 수준의 우울과 관련이 있는 것으로 나타나 치매노인 가족돌봄자의 우울 관리 필요성을 강조하고 있다[35].

반면, 치매태도는 83.03점으로 동일한 도구로 측정한 선행연구[15,26] 결과인 80.50점, 80.65점보다 더 높게 나타나, 본 연구의 가족돌봄자의 치매태도가 더 긍정적이었다. 그리고 중앙치매센터 연차보고서[3]의 대국민 치매태도 점수를 환산한 값은 83.02점으로 본 연구와 유사하였다. 또한, 치매태도가 긍정적일수록 돌봄부담이 낮아졌으며, 선행연구 역시 치매태도가 긍정적일수록 돌봄에 대한 만족감이 높아지고 돌봄부담이 낮아지는 결과를 보였다[15,16]. 즉, 긍정적인 치매태도는 돌봄부담을 경감시키는 주요 변수로 볼 수 있다. 이에 긍정적인 치매태도를 형성시키는 것이 필요하며, 치매노인에 대한 두려움, 불안, 부정적 고정관념 등을 감소시키는 다양한 대처 기술을 교육하는 것이 방안이 될 수 있을 것이다.

회복탄력성은 돌봄부담, 돌봄시간, 행동심리증상, 우울과 유의한 음의 상관관계가 있는 것으로 나타났고, 우울과는 특히 높은 음의 상관관계를 보였다. 그리고 선행연구에서도 회복탄력성은 돌봄부담과 유의한 음의 상관관계에 있었다[17,36]. 또한, 가족돌봄자를 대상으로 한 연구는 아니지만, 지역사회 노인을 대상으로 한 연구에서 회복탄력성과 우울은 음의 상관관계에 있었다[37]. 이처럼 회복탄력성 역시 돌봄부담을 감소시키는 주요 변수로 볼 수 있다. 게다가, 스트레스 상황에 대한 긍정적 적응 능력을 의미하는 회복탄력성은 훈련을 통해 증진시킬 수 있다[17,38]. 따라서, 향후 가족돌봄자 프로그램 제공 시 회복탄력성 강화 방안을 포함하는 것이 필요하며, 이는 우울 감소에도 효과가 있을 것이다[36,37].

돌봄부담의 주요 영향요인을 확인하기 위한 단계적 다중회귀분석 결과, 가족돌봄자의 돌봄부담은 행동심리증상, 우울, 돌봄시간, 회복탄력성 순으로 영향을 받는 것으로 나타났다. 즉, 치매노인의 행동심리증상이 심할수록, 가족돌봄자의 우울 정도가 높을수록, 돌봄시간이 많을수록, 회복탄력성 점수가 낮을수록 돌봄부담이 증가하였다. 선행연구에서도 이와 유사한 결과를 보였는데, 치매노인의 행동심리증상이 심할 때, 하루 돌봄시간이 17시간 이상일 때, 돌봄부담이 높은 것으로 나타났다[12]. 게다가, 행동심리증상이 과거에는 질병의 후기에 나타나는 것으로 여겨졌으나, 현재는 초기에도 흔히 나타나는 것으로 보고되고 있다[39]. 그리고 행동심리증상은 인지기능장애 보다 더 큰 돌봄부담을 유발하는 요인으로 알려져 있고[40], 사회적 비용도 증가시키므로[41] 행동심리증상을 초기 단계부터 잘 관리하는 것이 무엇보다 중요함을 알 수 있는 결과이다. 또한, 메타분석 결과에 따르면, 치매노인의 행동심리증상은 돌봄부담의 가장 큰 영향요인이었고, 돌봄시간 역시 주요 영향요인으로 나타났다[42].

우리나라 국가치매관리사업은 2008년 제1차 치매관리종합대책부터 시작되어 현재 제4차 치매관리종합대책(2021-2025)이 추진되고 있다. 그리고 십여 년이 넘는 기간 동안 전국에 치매안심센터가 설치되어 운영되고 있으며, 치매안심 요양병원에서는 치매전문 치료서비스를 제공하고 있다. 하지만, 가족돌봄자는 여전히 많은 돌봄부담을 호소하고 있으며, 지원이 충분하지 않음을 본 연구를 통해 확인할 수 있었다. 따라서 이제는 치매노인과 가족이 지역사회에서 안정적인 생활을 보장받을 수 있도록 돌봄부담 감소를 위한 구체적인 지원이 필요하다.

이에 치매안심센터에서는 프로그램 계획 시, 치매노인의 행동심리증상 조절, 가족돌봄자의 돌봄시간 감소, 정신건강 증진, 회복탄력성 향상을 우선 고려할 필요가 있다. 특히, 치매노인의 행동심리증상 조절에 도움이 되는 다양한 행동지향적, 인지지향적, 자극지향적 활동과 정서적 지원을 제공하는 프로그램을 확대할 필요가 있다[43-45]. 그리고 가족돌봄자에게는 치매노인의 행동심리증상을 이해하고 적절하게 대처할 수 있도록 다양한 선행연구 결과를 참고한 관련 교육을 제공할 필요가 있다[46,47]. 또한, 정신건강 및 회복탄력성 증진을 위해 여가생활지원과 건강관리를 위한 사회적 지원을 제공하고, 치매노인이 지역사회에서 안심하고 살아갈 수 있도록 치매친화적인 환경을 조성해야 할 것이다[9]. 특히, 우울 증상이 있는 가족돌봄자에게 맞춤형 사례관리, 서비스 코디네이션 등을 강화한 사업을 추가로 제공하여 기존의 조기검진 위주 사업에서 더 나아가 지역사회 치매관리사업의 허브 역할을 할 수 있어야 한다.

본 연구의 결과는 다음과 같은 중요한 의의를 지닌다. 먼저, 서울시 전체 25개 치매안심센터 중 20%에 해당하는 5개 센터가 연구에 참여함으로써, 표본의 대표성을 일정 부분 확보하였다. 이를 통해 보다 포괄적인 관점을 제시할 수 있었다는 점에서 의미가 크다. 또한, 돌봄 부담의 영향 요인을 분석함에 있어 부정적 요인 뿐만 아니라 긍정적 요인도 함께 고려하였다. 이는 기존 연구들이 주로 부정적인 측면에 초점을 맞추어 왔던 것과 차별화되는 점으로, 돌봄 부담을 줄일 수 있는 보호적인 요소들을 확인했다는 데 의의가 있다.

그러나 본 연구는 몇 가지 제한점을 가지고 있다. 첫째, 편의 표본 추출 방식을 사용하여 조사가 이루어졌다는 점에서, 연구 결과를 전국적으로 일반화하는 데 한계가 있다. 추후 연구에서는 더 다양한 지역과 환경에서의 검토가 필요할 것이다. 둘째, 치매 노인들이 겪고 있는 구체적인 치매 관련 세부 증상이 충분히 고려되지 않았다는 점도 주의해야 한다. 치매의 증상은 매우 다양하고, 각 증상에 따라 돌봄자의 부담 정도가 달라질 수 있는데, 본 연구에서는 이러한 증상별 특성을 구체적으로 반영하지 못했다. 따라서, 연구 결과를 해석할 때는 이러한 한계를 염두에 두고 접근할 필요가 있다. 이에 향후 연구에서는 치매 증상의 세부 유형에 따른 돌봄 부담의 차이점을 보다 면밀히 분석하는 것이 필요할 것이다.

## 결론

본 연구는 지역사회 치매안심센터에 등록되어 센터의 서비스를 이용하며 치매 노인을 돌보고 있는 가족돌봄자의 돌봄부담에 영향을 미치는 요인을 분석하기 위해 수행되었다. 연구 결과, 치매노인의 가족돌봄자에게서 행동심리증상, 우울, 돌봄시간, 회복탄력성이 돌봄 부담에 통계적으로 유의한 영향을 미치는 요인으로 확인되었다. 이러한 결과는 지역사회 내 치매안심센터의 운영에 있어 유용한 참고자료로 활용될 수 있을 것이다. 다만, 본 연구에서 다룬 요인 외에도 돌봄부담에 영향을 미칠 수 있는 다양한 가능성이 존재하므로, 향후 연구에서는 더 다양한 대상자 특성을 고려해야 할 필요가 있다. 또한, 이번 연구는 주로 도심 지역의 가족돌봄자를 대상으로 진행되었기 때문에, 앞으로는 다양한 지역사회 환경에 있는 가족돌봄자를 대상으로 한 연구가 필요하다. 마지막으로, 치매 진단 초기부터 사망에 이르기까지 전 과정을 더 정확하게 측정할 수 있는 세분화된 평가도구를 개발하고, 이를 기반으로 개별화된 가족 지원 프로그램을 개발하여 그 효과를 검증하는 후속 연구를 제언한다.

## Figures and Tables

**Table 1.** Mean Differences in Care Burden according to Family Caregiver and Care Receiver Factors (N = 234)

Variables	Categories	n (%)	Care burden		
		M ± SD	M ± SD	t or F	*p*
Family caregiver factors					
Sex	Men	40 (17.1)	22.35 ± 10.94	-0.43	.668
	Women	194 (82.9)	20.09 ± 9.72		
Age (yr)		65.80 ± 12.83			
	<65	95 (40.6)	22.86 ± 9.48	1.20	.302
	65∼75	76 (32.5)	24.22 ± 9.73		
	>75	63 (26.9)	21.60 ± 10.83		
Education	None	17 (7.3)	28.53 ± 9.65	2.06	.086
	Elementary school	33 (14.1)	20.88 ± 10.23		
	Middle school	30 (12.8)	24.70 ± 10.62		
	High school	83 (35.5)	22.30 ± 10.06		
	More than college	71 (30.3)	22.65 ± 9.14		
Religion	No	80 (34.2)	22.63 ± 10.02	-0.38	.707
	Yes	154 (65.8)	23.14 ± 9.94		
Subjective health status	Poor^a^	72 (30.8)	26.92 ± 9.87	10.65^*^	<.001
	Moderate^b^	93 (39.7)	22.43 ± 9.33		a > b,c
	Good^c^	69 (29.5)	19.57 ± 9.54		
Relationship with care receiver	Spouse	123 (52.6)	23.54 ± 10.40	0.58	.561
	Son or daughter	82 (35.0)	22.65 ± 9.48		
	Others	29 (12.4)	21.45 ± 9.42		
Living together	Yes	192 (82.1)	23.76 ± 10.09	2.64	.009
	No	42 (17.9)	19.33 ± 8.47		
Hours of caregiving (hr/day)		13.62 ± 7.80			
	≤12	107 (45.7)	20.24 ± 9.00	3.96	<.001
	>12	127 (54.3)	25.26 ± 10.20		
Duration of caregiving (yr)		4.26 ± 3.36			
	≤5	168 (71.8)	22.46 ± 10.04	0.60	.662
	>5	66 (28.2)	24.26 ± 9.69		
Care receiver factors					
Sex	Men	119 (50.9)	22.96 ± 9.72	-0.01	.990
	Women	115 (49.1)	22.97 ± 10.22		
Age (yr)		81.74 ± 7.71			
	< 75	37 (15.8)	22.70 ± 7.24	0.18	.829
	75 ∼ 84	116 (49.6)	23.37 ± 10.87		
	> 85	81 (34.6)	22.51 ± 9.69		
Education	None	36 (15.4)	23.50 ± 10.98	1.17	.323
	Elementary school	73 (31.2)	24.48 ± 9.81		
	Middle school	41 (17.5)	21.27 ± 8.47		
	High school	55 (23.5)	21.36 ± 10.55		
	More than college	29 (12.4)	23.93 ± 9.61		
BPSD		9.66 ± 7.50			
	No	20 (8.5)	11.70 ± 7.23	-5.63	<.001
	Yes	214 (91.5)	24.02 ± 9.52		

M = Mean; SD = Standard deviation; BPSD = Behavioral and psychological symptoms of dementia.

^*^Scheffé test.

**Table 2.** Family Caregivers’ Care Burden, Depression, Dementia Attitudes, and Resilience (N = 234)

Variables		Min	Max	M ± SD
Care Burden	Total	0	48	22.97 ± 9.95
	Personal strain	0	36	17.12 ± 8.56
	Role strain	0	12	5.81 ± 2.64
Depression		0	26	7.07 ± 5.99
Dementia attitudes		44	126	83.03 ± 16.20
Resilience		6	30	19.55 ± 4.72

Min = Minimum; Max = Maximum; M = Mean; SD = Standard deviation.

**Table 3.** Relationships among Major Research Variables (N = 234)

Variables	1	1-1	1-2	2	3	4	5	6
	*r (p)*							
1. Care burden	1	-	-	-	-	-	-	-
1-1. Personal strain	.97 (< .001)	1	-	-	-	-	-	-
1-2. Role strain	.62 (< .001)	.42 (< .001)	1	-	-	-	-	-
2. Hours of caregiving	.28 (< .001)	.33 (< .001)	.02 (.751)	1	-	-	-	-
3. BPSD	.56 (< .001)	.57 (< .001)	.28 (< .001)	.10 (.129)	1	-	-	-
4. Depression	.52 (< .001)	.51 (< .001)	.30 (< .001)	.18 (.006)	.42 (< .001)	1	-	-
5. Dementia attitudes	-.25 (< .001)	-.25 (< .001)	-.11 (< .001)	-.18 (.006)	-.13 (.051)	-.21 (.001)	1	-
6. Resilience	-.34 (< .001)	-.32 (< .001)	-.24 (< .001)	-.11 (.090)	-.22 (< .001)	-.41 (< .001)	.38 (< .001)	1

BPSD = Behavioral and psychological symptoms of dementia.

**Table 4.** Multiple Regression Analysis of the Care Burden of Family Caregivers (N = 234)

Variables	B	SE	β	*t*	*p*
(Constant)	16.63	2.77		6.00	< .001
BPSD	0.54	0.07	.41	7.52	< .001
Depression	0.43	0.10	.26	4.46	< .001
Hours of caregiving	0.23	0.06	.18	3.71	< .001
Resilience	-0.25	0.11	-.12	-2.31	.022
F = 47.76 (*p* < .001), R^2^ = .46, Adjusted R^2^ = .45, Durbin-Watson = 1.96					

SE = Standard error; BPSD = Behavioral and psychological symptoms of dementia.

## Data Availability

The datasets generated and/or analyzed during this study are not publicly available to protect participant confidentiality but are available from the corresponding author upon reasonable request.

## References

[1] Central Dementia Center (2023). Korean dementia observatory 2022 [Internet]. https://www.nid.or.kr/info/dataroom_view.aspx?bid=257.

[2] World Health Organization (2017). The global action plan on the public health response to dementia 2017-2025 [Internet]. https://www.who.int/publications/i/item/global-action-plan-on-the-public-health-response-to-dementia-2017---2025.

[3] Central Dementia Center (2022). National institute of dementia annual report 2021 [Internet]. https://www.nid.or.kr/info/dataroom_view.aspx?bid=239.

[4] Yoo J, Bae H, Lee Y, Lim J, Kim S, Jung K (2018). Public policy for the older adults with dementia and their caregivers [Internet]. http://repository.kihasa.re.kr/handle/201002/31310.

[5] Heo S, Kim S, Cheon S, Jang S, Park K (2021). A Factors affecting the caregiver burden of family caregivers in patients with degenerative brain disease. Social Science Research Review Kyungsung University.

[6] Kim E, Park H (2019). Factors associated with burden of family caregivers of home-dwelling elderly people with dementia: a systematic review and meta-analysis. Korean Journal of Adult Nursing.

[7] Kim J, Ryu W, Choi Y (2018). The effect of caregiver burden on depression of dementia family caregivers: the moderating role of perceived public support. Korean Journal of Gerontological Social Welfare.

[8] Vitaliano PP, Zhang J, Scanlan JM (2003). Is caregiving hazardous to one's physical health? A meta-analysis. Psychological Bulletin.

[9] Choi J, Lee J (2018). Factors influencing the family burden of taking care of elderly with dementia. Journal of Korean Home Management Association.

[10] Kwon S, Lee H, Lee J (2015). The effects of care hours on family caregiver’s burden for the elderly with dementia: focusing on the moderating effects of the attachment. Social Science Research Review.

[11] Lee HJ, Lee JW, Lee JY (2015). Family caregiver's burden for the elderly with dementia: moderating effects of social support. Journal of Social Science.

[12] Lim KC (2019). Influencing factors on care burden among family caregivers for elders with dementia: focusing on family caregivers using a support center for dementia. The Journal of Korean Academic Society of Nursing Education.

[13] Hong SJ, Ko E, Choi M, Sung NJ, Han MI (2022). Depression, anxiety and associated factors in family caregivers of people with dementia. Journal of Korean Neuropsychiatric Association.

[14] Park J, Lee W, Jeong H, Hong N, Bae B, Yim H (2020). Characteristics of depressive symptoms in middle-aged family members of dementia patients: 2017 Korea Community Health Survey. Epidemiology and Health.

[15] Choi J, Jeong H, Park J, Kim T, Lee D, Lee D (2015). Factors associated with the attitudes toward dementia in community caregivers: results from the nationwide survey on dementia care in Korea. Journal of Korean Geriatric Psychiatry.

[16] Hong J, Jung D (2020). The impact of dementia knowledge and attitude on caregiving appraisal among caregivers of older adults with dementia using dementia care centers. Journal of Korean Gerontological Nursing.

[17] Paik JE (2021). A study on resilience of dementia elderly and their family. Journal of Next-generation Convergence Technology Association.

[18] Kim Y, Byun S (2020). Effects of the burden on the quality of life, stress, and depression of the family caring for the elderly with dementia. The Journal of Humanities and Social Sciences 21.

[19] Sung M, Yi M, Lee D, Jang H (2013). Overcoming experiences of family members caring for elderly patients with dementia at home. Journal of Korean Academy of Nursing.

[20] Lee Y, Park M (2016). Factors influencing caregiving satisfaction among family caregivers of patients with dementia. Journal of Korean Gerontological Nursing.

[21] Faul F, Erdfelder E, Lang AG, Buchner A (2007). G*Power 3: a flexible statistical power analysis program for the social, behavioral, and biomedical sciences. Behavior Research Methods.

[22] Kwon J, Hong GRS (2021). Influence of self-care on burnout in primary family caregiver of person with dementia. Journal of Korean Academy of Nursing.

[23] Kim HJ, Choi KH, Kim SH, Cummings JL, Yang DW (2016). Validation study of the Korean version of the brief clinical form of the neuropsychiatric inventory. Dementia and Geriatric Cognitive Disorders Extra.

[24] Zarit SH, Orr NK, Zarit JM (1985). The hidden victims of Alzheimer’s disease: families under stress.

[25] Bédard M, Molloy DW, Squire L, Dubois S, Lever JA, O'Donnell M (2001). The Zarit burden interview: a new short version and screening version. The Gerontologist.

[26] Kim K, Kwak G, Kim G, Kim M, Kim B, Kim S (2011). The condition of the elderly with dementia [Internet]. https://e-learning.nhi.go.kr/oer/view/oerCntnsView.do?id=97656&cid=NB000120061201100031424&oid=0000000006&mid=view.

[27] Spitzer RL, Kroenke K, Wiliams JB (1999). Validation and utility of a self-report version of PRIME-MD: the PHQ primary care study. The Journal of the American Medical Association.

[28] Park SJ, Choi HR, Choi JH, Kim K, Hong J (2010). Reliability and validity of the Korean version of the Patient Health Questionnaire-9 (PHQ-9). Anxiety and Mood.

[29] O'Connor ML, McFadden SH (2010). Development and psychometric validation of the dementia attitudes scale. International Journal of Alzheimer’s Disease.

[30] Smith BW, Dalen J, Wiggins K, Tooley E, Christopher P, Bernard J (2008). The brief resilience scale: assessing the ability to bounce back. International Journal of Behavioral Medicine.

[31] Choi N, Leach SM, Hart JM, Woo H (2019). Further validation of the brief resilience scale from a Korean college sample. Journal of Asia Pacific Counseling.

[32] Kang CM, Kim JS, Jeong JH (2016). Factors influencing quality of caregiving by caregivers for elders with dementia. Journal of Korean Academy of Community Health Nursing.

[33] Lee HK, Kim SY (2019). Factors influencing the caring burden of families with dementia in a community. Journal of the Korean Applied Science and Technology.

[34] Wolfs CA, Kessels A, Severens JL, Brouwer W, de Vugt ME, Verhey FR (2012). Predictive factors for the objective burden of informal care in people with dementia: a systematic review. Alzheimer Disease & Associated Disorders.

[35] Schulz R, Sherwood PR (2008). Physical and mental health effects of family caregiving. American Journal of Nursing.

[36] Gilsenan J, Gorman C, Shevlin M (2022). Explaining caregiver burden in a large sample of UK dementia caregivers: the role of contextual factors, behavioural problems, psychological resilience, and anticipatory grief. Aging & Mental Health.

[37] Jang M, Hong CH, Chang KJ, Han C, Jeon SW, Roh HW (2016). The relationship between late-life depression and resilience. Journal of Korean Geriatric Psychiatry.

[38] Kim JH (2019). Resilience: the power of mental strength to turn trials into good luck.

[39] Angeles RC, Berge LI, Gedde MH, Kjerstad E, Vislapuu M, Puaschitz NG (2021). Which factors increase informal care hours and societal costs among caregivers of people with dementia? A systematic review of Resource Utilization in Dementia (RUD). Health Economics Review.

[40] Lyketsos CG, Carrillo MC, Ryan JM, Khachaturian AS, Trzepacz P, Amatniek J (2011). Neuropsychiatric symptoms in Alzheimer’s disease. Alzheimer’s & Dementia.

[41] Schulz R, O’Brien AT, Bookwala MS, Fleissner K (1995). Psychiatric and physical morbidity effects of dementia caregiving: prevalence, correlates, and causes. The Gerontologist.

[42] Kim EK, Park H (2019). Factors associated with burden of family caregivers of home-dwelling elderly people with dementia: a systematic review and meta-analysis. Korean Journal of Adult Nursing.

[43] Kwon MH, Lee JS (2017). The effect of nonpharmacologic interventions on behavioral and psychological symptoms of dementia: a meta-analysis. The Journal of the Korea Contents Association.

[44] Kang KJ, Kang MJ (2021). Effects of horticultural therapy on Korean elderly with dementia: a meta-analysis. Journal of Korean Academy of Psychiatric and Mental Health Nursing.

[45] Kim Y, Cho E (2022). Non-pharmacological intervention for wandering behavior in dementia: a systematic review and meta-analysis. Journal of Korean Gerontological Nursing.

[46] Park H, Gang M (2015). The analysis of the educational programs for family caregivers to manage problematic behaviors among patients with dementia. Keimyung Journal of Nursing Science.

[47] Lee SA, Kim HS (2017). Effects of a dementia family education program for dementia recognition, burden, and depression in caregivers of elders with dementia. Journal of Korean Academy of Psychiatric and Mental Health Nursing.

